# The US4ABL Strategy: A Systematic Ultrasound-Guided Approach for Left Atrial and Ventricular Ablation Procedures

**DOI:** 10.3390/jcm14010103

**Published:** 2024-12-27

**Authors:** Alexandru Gabriel Bejinariu, Nora Augustin, Maximilian Spieker, Carsten auf der Heiden, Stephan Angendohr, Moritz Höckmann, Lukas Clasen, Stefan Hartl, Hisaki Makimoto, Lucas Busch, Malte Kelm, Obaida Rana

**Affiliations:** 1 Division of Cardiology, Pulmonology and Vascular Medicine, Medical Faculty, Heinrich-Heine University, 40225 Düsseldorf, Germany; 2 Department of Cardiology, Rhythmology and Angiology, Josephs-Hospital Warendorf, Academic Teaching Hospital, University of Münster, 48231 Warendorf, Germany; 3 Department of Electrophysiology, Alfried Krupp Hospital, 45121 Essen, Germany; 4 Department of Medicine, Witten/Herdecke University, 58455 Witten, Germany; 5 Data Science Center/Cardiovascular Center, Jichi Medical University, Yakushiji 3311-1, Shimotsuke-City 329-0498, Japan; 6 CARID, Cardiovascular Research Institute Düsseldorf, Medical Faculty, Heinrich-Heine-University, 40225 Düsseldorf, Germany

**Keywords:** ultrasound, US4ABL, ablation, complication

## Abstract

**Background**: The safety and efficacy of electrophysiological (EP) procedures using ultrasound (US) guidance are being increasingly studied. We investigated if a systematic workflow with ultrasound guidance (the US4ABL), comprising four steps (transesophageal echocardiography (TEE) for left atrial thrombus exclusion, US of the groin vessels to guide femoral access, TEE-aided transseptal puncture, and transthoracic echocardiography (TTE) for exclusion of pericardial tamponade after the procedure), reduces the number of complications and fluoroscopy duration and dose. **Methods**: A total of 212 consecutive patients underwent left-sided ablations using the US4ABL workflow and were compared to a group of 299 patients who underwent the same type of ablations using post-procedural TTE to exclude tamponade (standard group: venous and/or arterial access by palpation and fluoroscopy, and pressure guided transseptal puncture). Complications, procedural duration, fluoroscopy duration, and dose were compared. **Results**: The cohort included 511 patients (42% female); 43.8% of patients suffered from paroxysmal atrial fibrillation (AF), 35.4% presented with persistent AF, 10.7% underwent the procedure was for atrial tachycardia, and 10% of patients had premature ventricular contractions. The complication rate in the US4ABL group was lower compared to the standard group: 0 complications vs. 11 complications (3.7%, mainly vascular and pericardial), respectively (*p* = 0.005). The procedure times were lower in the US4ABL group (*p* < 0.01), whereas the fluoroscopy time and dose did not differ significantly. **Conclusions**: A fully ultrasound-guided (US4ABL) workflow for left atrial and ventricular electrophysiology procedures reduces the complication rate and the procedure time.

## 1. Introduction

Left-sided ablation procedures are the cornerstone of interventional therapy in the management of most heart rhythm disorders (i.e., atrial fibrillation, atrial tachycardias, accessory pathways, premature ventricular contractions, ventricular tachycardia). These interventions are accompanied by significant challenges, as they carry a considerable risk of complications that can impact patient outcomes [1]. As the global burden of cardiac diseases continues to rise, the imperative to refine these procedures and minimize associated complications has never been more critical.

Therefore, the use of ultrasound (US) during these procedures has gradually increased. However, adequately powered randomized controlled trials regarding US-guided femoral or transseptal access are lacking. As a result, the recommendations from the current guidelines arise from observational data.

The available literature focuses on the advantages of US in the electrophysiology (EP) laboratory, considering a single step of the ablation procedure: exclusion of left atrial thrombi [2,3], femoral access [4,5,6], transseptal puncture (TSP) [7,8], and pericardial effusion rule-out after sheath removal [9,10]. Moreover, in some studies transesophageal echocardiography (TEE) was performed twice for the first and third steps [7,11]. Therefore, data concerning the safety of ablations using a systematic US-guided workflow are lacking.

**Figure 1:**
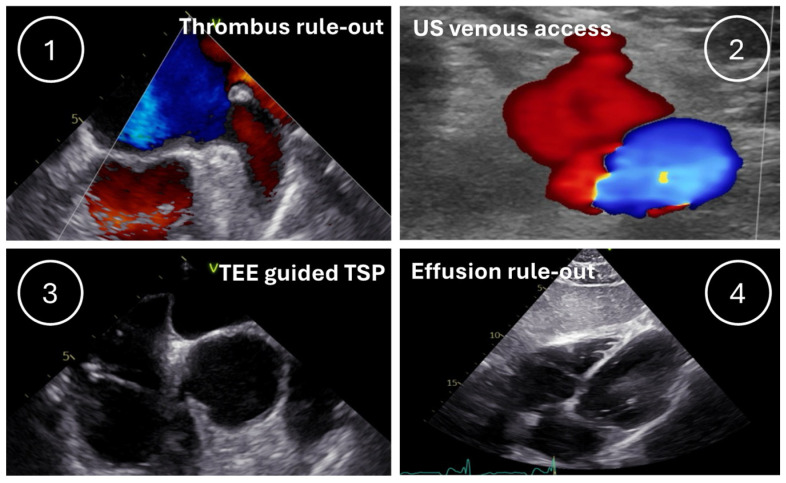
The UltraSound-guided „4“ ABLation strategy (US4ABL): 4 consecutive steps for left-sided ablations. Step 1: left atrial thrombi rule-out using TEE; Step 2: US-guided femoral access; Step 3: US-guided transseptal puncture; Step 4: pericardial effusion rule-out using TTE. US—ultrasound; TEE—transesophageal echocardiography; TTE—transthoracic echocardiography; TSP—transseptal puncture.

We investigated whether a systematic workflow with US guidance, including four steps (1. TEE for left atrial thrombus exclusion, 2. US of the groin vessels to guide femoral access, 3. TEE-aided transseptal puncture, and 4. transthoracic echocardiography (TTE) for exclusion of pericardial tamponade after the procedure), has an influence on the number of complications and fluoroscopy duration and dose (Figure 1).

## 2. Materials and Methods

### 2.1. Ethics

The local ethics committee of the Heinrich Heine University Düsseldorf approved the study (Number 2019-555_4). The study complies with the Declaration of Helsinki; all study participants gave their informed written consent.

### 2.2. Population

Consecutive patients undergoing left atrial or left ventricular (antegrade approach with TSP) procedures at our institution from September 2021 to February 2023, employing the US4ABL strategy, were included prospectively (the US4ABL group). In a second step, we compared this group to a second group of consecutive patients who had undergone the same type of procedures between 2018 and 2020 (the standard group). The primary outcome measure was the occurrence rate of major complications during or within the first 48 h after the procedure, including major groin complications (false aneurysms, arterio-venous fistula, hematoma as defined by a BARC type 2 criteria or higher [12]), pericardial tamponade, stroke, and death. The secondary outcome measure was the overall procedure duration, fluoroscopy time, and fluoroscopy dose.

Patients presenting with paroxysmal atrial fibrillation (AF), persistent AF, atrial tachycardia (AT), or premature ventricular contractions (PVC) with a history of failed antiarrhythmic therapy (class I and III antiarrhythmics) were included if they were older than 18 years and provided written informed consent prior to their inclusion. The exclusion criteria were contraindications to oral anticoagulants, thrombus in left atrium on TEE before the procedure, or valvular AF (in the case of left atrial procedures). The ablations for paroxysmal and persistent AF included only first-do procedures. The procedures were indicated according to the guideline recommendations from the European Society for Cardiology (ESC) [2,13].

We recorded and analyzed relevant pathologies, treatments, and epidemiological data.

### 2.3. Procedural Workflows

All ablation procedures were performed under intravenous sedation, using a combination of midazolam, propofol, and piritramide, if required. As per institutional procedural standards, heart rate, oxygen saturation, and non-invasive blood pressure readings were monitored during the procedure. Oral anticoagulation was continued until the evening before the procedure and resumed the next evening, after the intervention (after excluding pericardial tamponade), using a minimally interrupted protocol [14].

The objectives depended on the type of procedure and were as follows:

1. In pulmonary vein isolations (PVIs), a wide-area circumferential ablation was performed (ablation index guided), the procedural objective was the disappearance of the PV signal recorded on a circular mapping catheter or high-density mapping catheter (HDMC). The ablation was performed using the CLOSE protocol [15]. Briefly, we used a minimum contact force of 5 g, aiming to reach 10–20 g, 50 W irrespective of the anterior–posterior segment, with an ablation index target of 400 posterior and 550 anterior.
2. In left atrial tachycardias, mapping with a HDMC was performed, and the final lesion sets were at the operator’s discretion, with an objective of non-inducibility.
3. In left ventricular cases, mapping and ablation were performed with the ablation catheter, with the objective of the complete elimination of the PVC.

**Figure 2:**
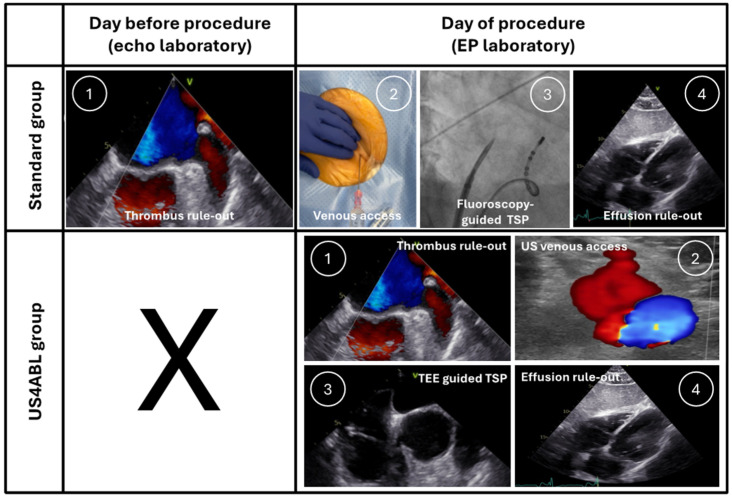
Comparison between the standard workflow and the US4ABL strategy. US—ultrasound; TEE—transesophageal echocardiography; TSP—transseptal puncture.

All procedures were performed by two experienced electrophysiologists. The group-specific workflows are presented in the following sections (Figure 2).

#### 2.3.1. Standard Group

Preprocedural TEE, including a three-dimensional (3D) assessment, was performed in the echocardiography laboratory up to 24 h prior to the EP procedure in all patients, to rule out atrial thrombi and significant valve disease.

After obtaining femoral venous access in the right groin by palpation only and placing three short sheaths (8F, 8F, and 6F), an octapolar 6F steerable diagnostic catheter (Inquiry, Abbott Medical, Green Oaks, IL, USA) was placed in the coronary sinus. The TSP was then performed fluoroscopically and pressure-guided (double TSP in left atrial procedures and single TSP in left ventricular procedures) using SL1 Swartz sheaths and a BRK-1 needle (Abbott Medical, Green Oaks, IL, USA). In left ventricular procedures, the SL1 sheath was then exchanged for a steerable Agilis NxT sheath (Abbott Medical, Green Oaks, IL, USA). A 3D-electroanatomical mapping system was used in all cases (CARTO3, Biosense Webster, Diamond Bar, CA, USA), in combination with a contact force sensing ablation catheter (Thermocool SmartTouch SF, Biosense Webster, Diamond Bar, CA, USA).

After the ablation, pericardial effusion was ruled out by TTE in the EP laboratory. After sheath removal, a figure-of-8 suture was placed to achieve hemostasis. Following the ablation procedures, the patients were transferred to the ward and monitored for the next 2 days.

#### 2.3.2. US4ABL Group

The US4ABL strategy was developed to enhance safety and consists of four steps, each of which US-guided. The echocardiography machine employed in the EP laboratory was a Vivid T8 (GE Healthcare, Chicago, IL, USA).

The first step is the exclusion of thrombi in the left atrium using TEE, performed in the electrophysiological laboratory before obtaining venous access. Additionally, a full TEE examination was performed, to exclude significant valve disease. Extended 3D echocardiography data was not obtained and, furthermore, PFO and atrial shunts were only examined visually without special provocation tests. The echocardiographies were performed by experienced physicians in peri-interventional imaging. The TEE probe remains in the esophagus to guide the TSP later.

The second step is to gain venous femoral access (3 punctures) in the right groin using a linear transducer (range from 7.5 to 18 MHz). After placing 3 short sheaths (8F, 8F, and 6F), an octapolar steerable catheter (Inquiry, Abbott Medical, Green Oaks, IL, USA) was introduced in the coronary sinus.

The third step is to guide the TSP; the sheath and the transseptal needle were positioned in the fossa ovalis using fluoroscopy and TEE. To reach an ideal position, adjustments antero–posterior in 45° and superior–inferior in 90–110° were undertaken. The needle was used for puncturing the fossa ovalis and then the dilator with the sheath was carefully advanced using TEE. After removing the needle and dilator, the long sheath was left in the left atrium. After the second TSP and the exclusion of pericardial effusion, the TEE probe was removed (in the case of left atrial procedures).

The fourth step is to rule out pericardial effusion in the EP laboratory after the ablation procedure and sheath removal from the groin. After sheath removal, a figure-of-8 suture was placed to achieve hemostasis.

### 2.4. Statistics

SPSS statistical software version 26 (SPSS Inc., Chicago, IL, USA) was used for data analysis. Continuous variables are expressed as the median and interquartile range ([IQR]) if not normally distributed (otherwise mean ± standard deviation, if the normal distribution was documented), and categorical parameters are expressed as counts and percentages. The Shapiro–Wilk test was employed to assess for normal distribution. The Mann–Whitney U test was used for comparisons of continuous variables. Associations between categorical or discrete variables were assessed using the Pearson χ^2^ test or the Fisher exact test.

## 3. Results

We included 511 patients in the final analysis. The baseline characteristics of the cohort are summarized in Table 1. Most of the patients were of male sex (standard group 60%, US4ABL group 55%, *p* = 0.21). The median age was 69 (59–76) years in the standard group and 68 (58–75) years in the US4ABL group (*p* = 0.96). There was no significant difference among the baseline characteristics between the two groups, except for the indication for TSP (Table 1). There were no notable differences between the amount of procedures performed by each electrophysiologist: electrophysiologist 1 performed 49% of the procedures in the US4ABL group and 51% of the procedures in the standard group, whereas electrophysiologist 2 performed the remaining procedures.

The overall complication rates were 3.7% and 0.0% in the standard group and US4ABL group, respectively, which translated into a statistically significant result (*p* = 0.005).

There were seven major vascular access complications in the standard group, mainly represented by false aneurysms (n = 6) and one arterio-venous fistula (Table 2). All vascular access complications could be managed conservatively without the need for vascular surgery intervention, although prolonging the hospital stay.

Furthermore, four cardiac tamponades occurred in the standard group and were successfully managed by pericardial puncture. Two of these patients suffered TSP-related tamponades [16] (type C), whereas the other two patients suffered non-TSP-related tamponades (types D and F). One non-TSP-related tamponade occurred during a left ventricular ablation; the second non-TSP-related tamponade occurred during a left atrial ablation.

No further complications (i.e., stroke, clinical signs of esophageal injury, TEE-related complications) and no deaths occurred in either group.

The procedural data are shown in Table 3. The procedural duration differed significantly between the two groups; the US4ABL group showed shorter procedure times compared to the standard group. In terms of fluoroscopy times or dose–area product, there were no differences between both cohorts. The procedures driving the significant differences were the PVIs in both paroxysmal and persistent AF.

A subgroup analysis of procedural characteristics in patients with paroxysmal and persistent AF revealed that the procedural duration and fluoroscopy times were significantly lower in the US4ABL group, whereas the dose–area product did not differ (Table 4).

## 4. Discussion

We compared our fully US-guided US4ABL strategy to a standard approach with respect to safety and procedural parameters in left atrial and left ventricular procedures using an antegrade approach (TSP). The main findings are as follows: the US4ABL strategy (1) reduced the overall complication rate, (2) reduced the total procedural duration of AF ablations, and (3) did not have an impact on the fluoroscopy time and dose.

The main driver for the effect on the overall complication rate was the US-guided femoral access. There were also numerically fewer pericardial tamponades in the US4ABL group (n = 0). Since there were no complications following the TEE (in either group), the feasibility of a combined technique (exclusion of LAA thrombi in the EP laboratory immediately before starting the EP procedure and guidance of the TSP) is demonstrated for the first time with the present study.

Adequately powered randomized controlled trials (RCTs) for femoral venous access are lacking. The only RCT executed by Yamagata et al. [4] needed to be terminated due to lower-than-expected complication rates. Nonetheless, the authors showed that the US guidance improved the puncture times and led to fewer inadvertent arterial punctures. A further quasi-randomized study by Kupo et al. [6] demonstrated a decrease in the rate of both major and minor vascular complications, also supporting the routine use of US. The largest meta-analysis on US guidance for femoral venous access in EP procedures [17] showed, in more than 8000 patients, that this technique reduces vascular site complications, arterial complications, and puncture time. As a result, the European Heart Rhythm Association (EHRA) recommends, in its recently published consensus statement, the routine use of US-guided femoral access [9].

There is only scarce literature regarding TEE-guided TSP; RCT data are missing. Zuercher et al. [7] performed LA procedures using a workflow with TEE-guided TSP in 375 consecutive patients without any complications related to transseptal access. Moreover, the authors documented only 1 TEE-related complication (esophageal hematoma), proving an excellent safety profile. In the setting of PVI with pulsed-field ablation, our group showed that a TEE-aided TSP workflow is feasible and safe [18]. Similarly, our cohort benefited from the TEE by lowering the TSP-related complications without additional risks.

Recently, it has been demonstrated that, in left atrial appendage occlusion procedures, guidance by 3D intracardiac echocardiography (ICE) led to a high procedural success rate without increasing the complication rate [19]. Furthermore, 3D ICE was associated with a trend toward a reduction in leaks at follow-up. Therefore, this imaging modality needs to be investigated in a workflow like US4ABL.

The immediate pre-procedural exclusion of LAA thrombi in the electrophysiology laboratory has several advantages. Firstly, it unburdens the local echocardiography laboratory. Secondly, the electrophysiologist can directly assess the anatomy and prepare accordingly (i.e., appropriately bend the TSP needle, straightforward use of a radiofrequency needle in the case of a thickened fossa ovalis). Thirdly, if the local procedural workflow includes a TEE-guided TSP, the comfort of the patients is highly improved since they only undergo one TEE procedure.

To the best of our knowledge, our study is the first one to investigate a fully US-guided strategy consisting of (1) TEE for left atrial thrombus exclusion, (2) US of the groin vessels to guide femoral access, (3) TEE-aided transseptal puncture, and (4) TTE for exclusion of pericardial tamponade during and/or after the procedure. In our patient cohort, the US4ABL strategy reduced the overall complication rate to 0.0% and reduced the procedure time without any effect on fluoroscopy time and dose.

The critical limitation of this study is its partly retrospective design. Therefore, the next logical step is to design and execute an RCT to undoubtedly demonstrate the superiority of our strategy and recommend it on a large scale. A certain limitation is the lack of ICE in our workflow—mainly due to reimbursement issues—although it seems to have a good safety profile and enable zero-fluoroscopy workflows [20]. Another limitation when interpreting the results is the occurrence of one non-TSP-related cardiac tamponade during a left ventricular ablation. Furthermore, it is unclear why the US4ABL workflow shortens the procedure time.

## 5. Conclusions

This study shows that a combined, fully consecutive US-guided approach for left heart electrophysiology procedures reduced the overall complication rate and shortened the procedure time in atrial fibrillation ablations. An RCT is necessary to verify this workflow.

## Figures and Tables

**Table 1:** Baseline characteristics.

	Standard Group n = 299	US4ABL Group n = 212	*p* Value
Age, years	69 (59–76)	68 (58–75)	0.96
Male gender—n (%)	180 (60)	116 (55)	0.21
BMI—kg/m^2^ (IQR)	26 (24–30)	27 (24–31)	0.95
Indication for TSP—n (%)			0.01
Paroxysmal AF	115 (38.5)	109 (51.4)	
Persistent AF	100 (33.4)	81 (38.2)	
Left atrial tachycardia	40 (13.4)	15 (7.1)	
Left ventricular PVC	44 (14.7)	7 (3.3)	
Hypertension—n (%)	182 (61)	139 (66)	0.28
History of stroke—n (%)	33 (11)	30 (14)	0.29
Coronary artery disease—n (%)	90 (30)	71 (33.5)	0.41
Peripheral artery disease—n (%)	15 (5)	15 (7)	0.36
Heart failure—n (%)	61 (20)	39 (18)	0.21
Obstructive sleep apnea—n (%)	19 (6)	22 (10)	0.10
COPD—n (%)	16 (5)	19 (9)	0.11
CHA_2_DS_2_-VASc score (IQR)	3 (1–4)	3 (2–4)	0.51

IQR—interquartile range; BMI—body mass index; TSP—transseptal puncture; AF—atrial fibrillation; PVC—premature ventricular contraction; COPD—chronic obstructive pulmonary disease.

**Table 2:** Procedural complications.

	Standard Group n = 299	US4ABL Group n = 212	*p* Value
Major vascular access complication—n (%)	7 (2.3)	0 (0)	0.025
Cardiac tamponade—n (%)	4 (1.3)	0 (0)	0.091
Stroke—n (%)	0 (0)	0 (0)	
TEE-related complication—n (%)	0 (0)	0 (0)	
Esophageal injury—n (%)	0 (0)	0 (0)	
Total—n (%)	11 (3.7)	0 (0)	0.005

TEE—transesophageal echocardiography.

**Table 3:** Procedural characteristics.

	Standard Group n = 299	US4ABL Group n = 212	*p* Value
Procedural duration—min (IQR)	134 (100–180)	103 (75–135)	<0.01
Paroxysmal AF	121 (92–159)	95 (70–136)	<0.01
Persistent AF	142 (102–191)	105 (75–130)	<0.01
Left atrial tachycardia	135 (97–176)	105 (78–150)	0.15
Left ventricular PVC	165 (119–236)	165 (88–195)	0.36
Fluoroscopy time—min (IQR)	17 (12–25)	15 (11–22)	0.06
Dose–area product—cGy ⋅cm^2^ (IQR)	905 (564–1511)	853 (500–1721)	0.55

IQR—interquartile range; AF—atrial fibrillation; PVC—premature ventricular contraction.

**Table 4:** Procedural characteristics—AF subgroup.

	Standard Group n = 215	US4ABL Group n = 190	*p* Value
Procedural duration—min (IQR)	174 (139–217)	145 (106–179)	<0.01
Fluoroscopy time—min (IQR)	19 (12–27)	15 (11–22)	<0.01
Dose–area product—cGy⋅cm^2^ (IQR)	889 (572–1660)	828 (485–1698)	0.20

IQR—interquartile range; AF—atrial fibrillation.

## Data Availability

Raw data were generated at the University Hospital in Düsseldorf, Germany. The data that support the findings of this study are available from the principal investigator upon reasonable request.
